# Rigid High Temperature Heat-Shrinkable Polyimide Tubes with Functionality as Reducer Couplings

**DOI:** 10.1038/srep44936

**Published:** 2017-03-20

**Authors:** Deyan Kong, Xinli Xiao

**Affiliations:** 1 MIIT Key Laboratory of Critical Materials Technology for New Energy Conversion and Storage, School of Chemistry and Chemical Engineering, Harbin Institute of Technology, No. 92 West Dazhi Street, Harbin 150001, PRC

## Abstract

Flexible and semi-rigid heat-shrinkable tubes (HSTs) have been used in thousands of applications, and here rigid high temperature HSTs are reported for the first time. These rigid HSTs are prepared with shape memory polyimides possessing glass transition temperatures (*T*_*g*_s) from 182 to 295 °C, and the relationships between *T*_*g*_ and their molecular structures are studied. The polyimide HSTs (PIHSTs) can fix expanded diameters and shrink back to original diameters very well, and the mechanisms of their heat-shrinkage performance are discussed. Their differences from commercially available HSTs in heat-shrinkage are also analyzed. They can withstand low temperature of −196 °C, much lower than those of other HSTs. The PIHSTs can also connect subjects of different sizes by heat-shrinkage and then fix them upon cooling like reducer couplings, and the possible mechanisms of their reducer coupling effect are analyzed. With their unique characteristics, PIHSTs will expand the application areas of HSTs enormously.

Heat-shrinkable tubes (HSTs) are polymer tubes that can be expanded at high temperature and then maintain the expanded diameters at low temperature^1^,^2^,^3^. When reheated to the complete shrinkage temperatures (*T*_*s*_), HSTs will shrink back to their original sizes^4^,^5^. Compared with potting, taping or molding, HSTs are a valuable choice for insulating, protecting and sealing of wires, small parts or assemblies^6^,^7^. HSTs have been widely used in automobiles, electronics, computers, communications, aircrafts, petroleum, aerospace, military and so on. Literally, HSTs have been used in thousands of applications^2^. The proliferation of HST products has led to an ever-increasing number of market, and the annual yield of HSTs in China alone was more than 10 billion meters^2^,^8^. High temperature HSTs with *T*_*s*_ higher than 150 °C have been used in various applications such as electrical equipment and aerospace industries. However, the commercially available HSTs are flexible or semi-rigid, and there is no report about rigid high temperature HSTs until now^8^,^9^,^10^.

HSTs are in fact a typical commercial application of shape memory polymers (SMPs)^11^,^12^,^13^,^14^,^15^, and SMPs are smart polymers that can be programmed to fix temporary shapes and then recover to original shapes under external stimuli^16^,^17^,^18^,^19^,^20^. Many aspects of SMPs such as multi-stage, multi-functional, two-way reversible, composite, porous and fibrous SMPs have been flourishing in recent years^21^,^22^,^23^,^24^,^25^,^26^,^27^,^28^. However, the reports about SMPs have mainly focused upon their applications in textiles, biomedical devices, tissue engineering and aerospace^29^,^30^,^31^,^32^,^33^,^34^,^35^. HSTs have already possessed a rather mature market, and therefore they have attracted more attention from practical applications rather than scientific research.

*T*_*s*_ is the temperature at which HST can shrink back to its original size completely, and *T*_*s*_ is a crucial factor in determining their application areas. The HST will shrink spontaneously if its *T*_*s*_ is lower than the operating temperatures, while more heating is demanded and some damages may be caused to the environment if *T*_*s*_ is too high^18^,^23^,^28^. Commercially available HSTs are made of crystalline polymers and their *T*_*s*_s are closely related with their crystalline melting temperatures (*T*_*m*_s), as the melting of less-stable crystallites mainly determines the onset and end temperatures of their heat-shrinkage^36^,^37^,^38^,^39^. Completes heat-shrinkage of commercially available HST usually occurs at temperatures slightly higher than its *T*_*m*_, and HSTs prepared with polyvinyl chloride, polyolefin, polyvinylidene fluoride, silicone rubber and polytetrafluoroethylene possess *T*_*s*_s of about 105, 125, 175, 200 and 340 °C, respectively^9^,^10^. Although HSTs have been extensively used in many fields, their *T*_*s*_s are mainly confined around the above-mentioned temperatures. Therefore, high temperature HSTs with new *T*_*s*_s are expected to expand their application areas enormously.

In the current report, rigid high temperature HSTs prepared with polyimides are reported for the first time. Different from the reported flat films of shape memory polyimides, here we have developed polyimides into heat-shrinkable tubes^12^,^18^,^19^,^23^,^24^. Glass transition temperature (*T*_*g*_) is an important parameter for shape memory polyimide, and the polyimide heat-shrinkable tube (PIHST) can shrink back completely within several seconds at *T*_*g*_ + 20 °C. Therefore, *T*_*g*_ + 20 °C is employed as *T*_*s*_ of the PIHSTs. The PIHSTs can fix the expanded diameters and shrink back to original sizes very well, and the possible mechanisms of their heat-shrinkage performance are discussed. The diverse new types of shape memory polyimides in the current paper are different from the previously reported samples in both molecular structures and *T*_*g*_s. The shape memory polyimides possess *T*_*g*_s ranging from 182 to 295 °C, and the PIHSTs with corresponding *T*_*s*_s thus can expand the application areas of HSTs greatly. From the viewpoint of applications, low-temperature resistant property of HST is an important issue since some polymers will suffer from embrittlement at low temperatures^40^. Compared with the low temperature limits of about −40 to −80 °C for commercially available HSTs^3^,^8^, The PIHSTs can withstand low temperature of −196 °C and thus widen the operating temperature ranges of HSTs enormously.

Commercially available high temperature HSTs are unable to hold or fix heavy load since they are flexible or semi-rigid, and in some cases supporting frames are needed for the subjects concerned with HSTs. However, the supporting frames may lead to much inconvenience due to their extra weight and space occupied, especially for delicate or elaborate equipments such as advanced electrical and aerospace devices. Reducer couplings have been widely used in many applications such as petroleum and pharmacy transport devices, as they can offer flexibility and convenience in fixing subjects of different diameters without extra supporting frame^41^,^42^. Therefore, rigid HSTs that combine the advantages of both HSTs and reducer couplings can facilitate the application of HSTs greatly. The PIHSTs can also connect and fix different heavy subjects through heat-shrinkage, and the possible mechanisms of their reducer coupling effect were analyzed. With their unique properties, the rigid PIHSTs will find applications in many fields.

## Results.

### Molecular structures and morphologies of PIHSTs

**Figure 1:**
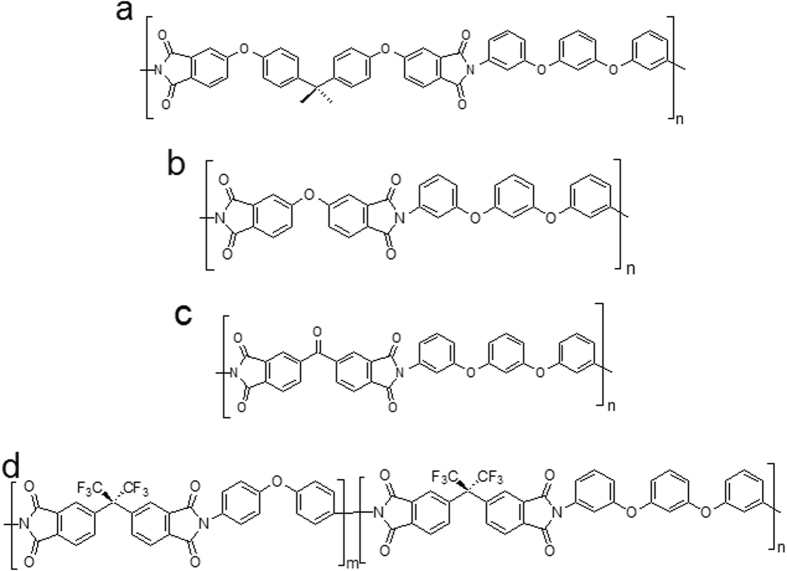
Molecular structures of the PIHSTs. (**a**) BPADA/BAB (PIHST01), (**b**) ODPA/BAB (PIHST02), (**c**) BTDA/BAB (PIHST03) and (**d**) 6FDA/ODA + BAB (PIHST04-PIHST08).

The rigid high temperature HSTs in the current report are prepared with shape memory polyimides, and the two-step polymerization process of these polyimides are shown in the supporting information as Figs S1–S4, respectively. IR results indicate that they are thoroughly imidized, as manifested in the supporting information (Fig. S5)^43^. These polyimides are different in molecular structures with three types of homopolyimides and five types of copolyimides, as shown in Fig. 1. The PIHSTs prepared with homopolyimides of BPADA/BAB, ODPA/BAB and BTDA/BAB are labeled as PIHST01, PIHST02 and PIHST03, respectively. Copolyimides represent a simple but effective way for structure modification to achieve desired properties, and they have attracted much attention in recent years^44^,^45^,^46^,^47^. The PIHSTs prepared with copolyimides of 6FDA/ODA + BAB are labeled as PIHST04, PIHST05, PIHST06, PIHST07 and PIHST08, whose molar ratios of ODA:BAB were 0.15:0.85, 0.30:0.70, 0.50:0.50, 0.70:0.30 and 0.88:0.12, respectively. Just like other copolyimides reported, the m/n ratios of ODA:BAB for the shape memory copolyimides are also similar to the specific molar ratios in the synthesis process, as the reactant monomers are not detected in the products^44^,^45^,^46^,^47^.

**Figure 2:**
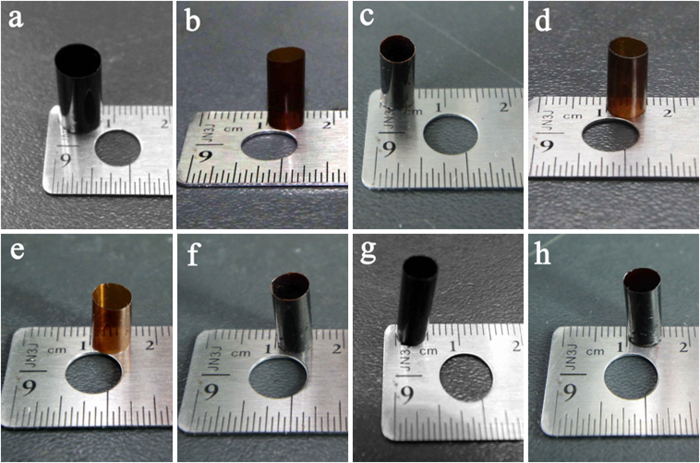
Typical images of the PIHSTs. (**a**)PIHST01, (**b**)PIHST02, (**c**)PIHST03, (**d**)PIHST04, (**e**)PIHST05, (**f**)PIHST06, (**g**) PIHST07 and (**h**)PIHST08.

The PIHSTs were obtained by evaporating their solutions in glass tubes, and the typical images of the PIHSTs with different diameters and wall thicknesses are shown in Fig. 2. It is demonstrated that the preparation method of PIHSTs is universal, as different types of shape memory homopolyimides and copolyimides have been made into tubes with this method. These photographs also indicate that the dimensions of PIHSTs are alterable, thus guaranteeing their prospects in applications since variable dimensions are needed to satisfy the demands of different practical environments.

### Thermomechanical and thermal properties of PIHSTs

**Figure 3:**
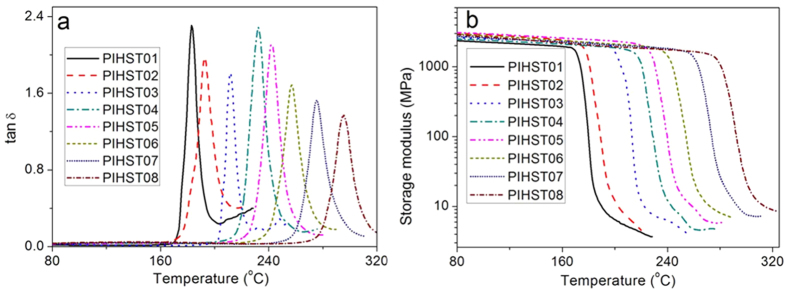
Thermomechanical properties of the PIHSTs. (**a**) *T*_*g*_s and (**b**) storage modulus versus temperature of the PIHSTs.

Thermomechanical properties of the polyimides were examined with DMA, and their *T*_*g*_s and storage modulus are shown in Fig. 3. *T*_*s*_ is important for HSTs since different application environments demand different *T*_*s*_s, and the PIHSTs possess different *T*_*s*_s corresponding to their *T*_*g*_s. It is well-known that more flexible molecular chain of polyimide will lead to lower *T*_*g*_, and the flexibility sequence of the homopolyimide chains is BPADA/BAB > ODPA/BAB > BTDA/BAB. Accordingly, PIHST01, PIHST02 and PIHST03 exhibit *T*_*g*_s of 182, 193 and 211 °C (Fig. 3a), respectively. The copolyimides of 6FDA/ODA + BAB are random copolyimides, as determined by the single *T*_*g*_ 45,46. The *T*_*g*_s of the copolyimide fall into the temperature range determined by the corresponding pure homopolyimides, and an increase in *T*_*g*_ is observed with the increase of ODA content. The copolyimides of PIHST04, PIHST05, PIHST06, PIHST07 and PIHST08 exhibit *T*_*g*_s of 232, 242, 257, 276 and 295 °C (Fig. 3a), respectively. Therefore, we have offered a convenient method to obtain shape memory polyimide with controllable *T*_*g*_ from 223 to 316 °C by copolymerization of 6FDA/ODA + BAB.

The relationship between *T*_*g*_ and content of the copolymers can be correlated with Equations such as Fox Equation, Gordon-Taylor Equation and Kwei Equation^48^,^49^. For the current shape memory copolyimides, the relationship between *T*_*g*_ and the content is expressed with Fox Equation as Equation 1:


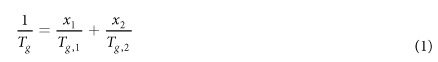


In Equation 1, *T*_*g*_ represents the *T*_*g*_ of 6FDA/BAB + ODA copolyimide, *T*_*g,1*_ and *T*_*g,2*_ indicate the *T*_*g*_ of pure 6FDA/BAB and pure 6FDA/ODA, while *x*_*1*_ and *x*_*2*_ refer to their corresponding mass fraction in 6FDA/BAB + ODA. It is observed that *T*_*g*_s of the copolyimides coincide with the reciprocal fit of Fox Equation very well, and the Adj. R-Square is 0.997, as shown in the supporting information (Fig. S6).

Storage modulus (*E*′) versus temperature of the PIHSTs are shown in Fig. 3b, and it is observed that there is a monotonic decrease of *E*′ with the increase of temperature at glassy state. There is a huge drop in *E*′ during glass transition, and then a plateau of *E*′ appears at rubbery state. *E*′ is of GPa at glassy state and then decreases to MPa at rubbery state. For example, *E*′ of PIHST03 at glassy state (*T*_*g*_ − 30 °C) and rubbery state (*T*_*g*_ + 20 °C) are 2.2 GPa and 7.2 MPa, and *E*′ of other PIHSTs are summarized in Table 1. Besides the high *T*_*s*_s, PIHSTs also possess high thermal stability as manifested by their TGA spectra in the supporting information (Fig. S7). The temperature at which 5% loss weight occurred is regarded as the decomposition temperature (*T*_*d*_), and *T*_*d*_s of PIHST01, PIHST02, PIHST03, PIHST04, PIHST05, PIHST06, PIHST07 and PIHST08 are 504, 547, 552, 555, 551, 557, 554 and 555 °C, respectively. These high *T*_*d*_s further confirmed that the PIHSTs are suitable for high temperature applications.

**Figure 4:**
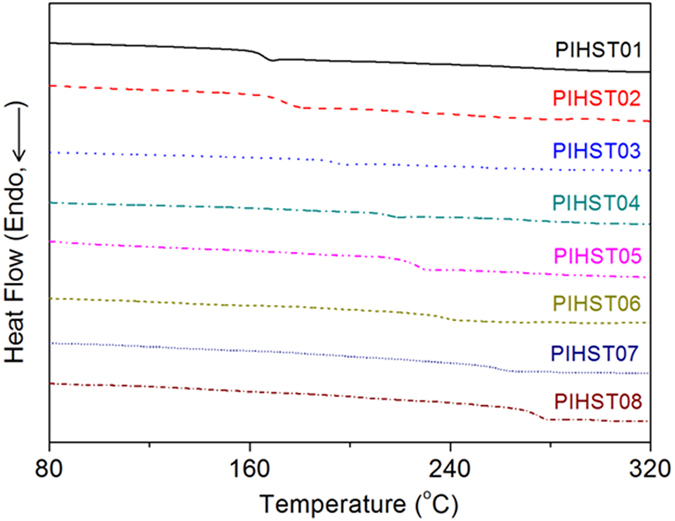
DSC spectra of the PIHSTs.

Commercially available HSTs possess clear evidence of crystallization and melting of crystallites in their differential scanning calorimetry (DSC) spectra, and their crystallinity decreased significantly after heat-shrinkage due to melting of crystallites and recoiling of chains^36^,^37^,^39^. There is no trace of crystallization or melting of crystallites in DSC spectra of PIHSTs (Fig. 4), indicating that they are amorphous. The heat-shrinkage of PIHSTs is caused by glass transition rather than melting of crystallites, and they are still amorphous after heat-shrinkage.

### Heat-shrinkable properties of PIHSTs

The shape memory performance of SMP is generally evaluated by shape fixity (*R*_*f*_) and shape recovery (*R*_*r*_), which indicate its ability to fix temporary shape and recover to original shape, respectively^20^,^22^,^50^. Heat-shrinkage is in fact a shape memory process, where the expanded diameter is the temporary shape and the shrunk diameter is the recovered shape. For the heat-shrinkage of PIHST, its *R*_*f*_ can be calculated with Equation 2.


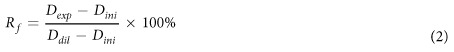


Here *D*_*ini*_, *D*_*dil*_ and *D*_*exp*_ indicate the diameters of initial, dilated and expanded PIHST, respectively. *R*_*r*_ of the heat-shrinkage can be calculated with Equation 3.


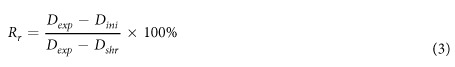


**Figure 5:**
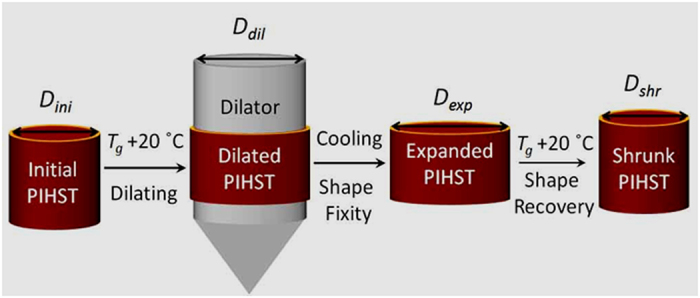
Schematic model of heat-shrinkage process of PIHSTs. *D*_*ini*_, *D*_*dil*_, *D*_*exp*_ and *D*_*shr*_ indicate the diameters of initial, dilated, expanded and shrunk PIHST, respectively.

Here *D*_*shr*_ indicates the diameter of shrunk PIHST, and the schematic model of heat-shrinkage process of PIHST are illustrated in Fig. 5. During practical operations, the expanded PIHST was removed from the oil bath set at *T*_*g*_ + 20 °C to room temperature of 25 °C within 1 second. Accordingly, the cooling temperature is 25 °C and the cooling rate is *T*_*g*_ − 5 °C/s.

**Figure 6:**
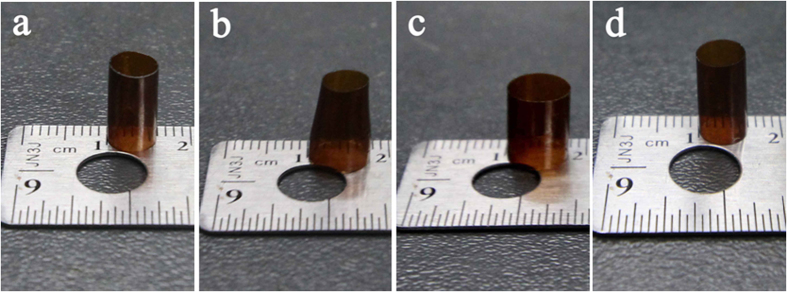
Heat-shrinkage process of PIHST. (**a**) Initial, (**b**) partially dilated, (**c**) fully expanded, and (**d**) shrunk states of PIHST04.

The heat-shrinkage process of PIHST04 is manifested in Fig. 6, and the *D*_*ini*_, *D*_*dil*_, *D*_*exp*_ and *D*_*shr*_ are 0.5832, 0.7559, 0.7532 and 0.5817 cm, respectively. Accordingly, PIHST04 exhibited *R*_*f*_ of 98.4% and *R*_*r*_ of 99.1%. The other PIHSTs can also fix expanded diameters and shrink back to original diameters nicely, and their *R*_*f*_s and *R*_*r*_s are summarized in Table 1. The polyimide lath also showed excellent shape memory performances in common bending deformation, as manifested in the supporting information (Fig. S8).

When commercially available HST is annealed or aged near its *T*_*s*_ (*T*_*m*_) for several minutes, the melting of crystallites usually cause it to fuse to the underlying materials as well. Therefore, they can be used only once in most cases. However, when PIHST is annealed or aged near its *T*_*s*_ (*T*_*g*_ − 30 °C to *T*_*g*_ + 20 °C) for longer time such as tens of minutes, there is no damage to it and the shape memory effect is not affected. Therefore, the PIHSTs can be recyclable.

**Figure 7:**
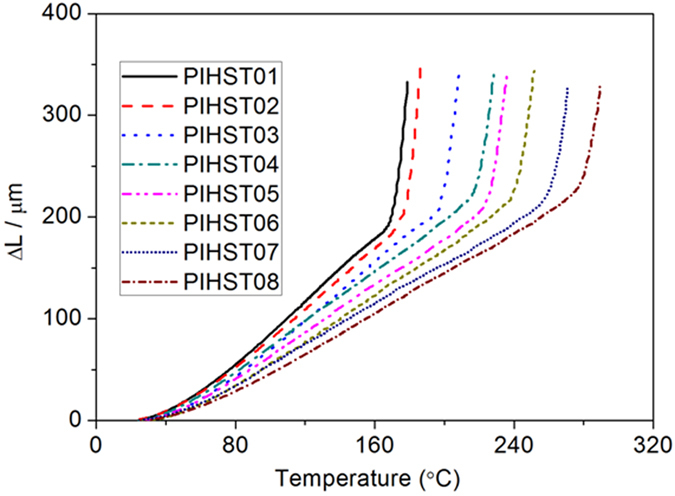
TMA spectra of the PIHSTs.

Thermal expansion coefficient (TEC) of the PIHSTs were characterized with thermal mechanical analyzer (TMA), as shown in Fig. 7. Due to the variation of TEC caused by temperature, the average TEC within certain temperature range is usually reported with Equation 4:


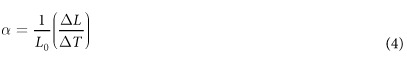


Here *L*_*0*_ is 15 mm, *∆T* = *T*_*2*_ − *T*_*1*_ with *T*_*2*_and *T*_*1*_ corresponding to 150 and 50 °C, and *∆L* = *L*_*2*_ − *L*_*1*_ with *L*_*2*_ and *L*_*1*_ indicating the sample lengths at 150 and 50 °C, respectively. The corresponding TECs of PIHST01, PIHST02, PIHST03, PIHST04, PIHST05, PIHST06, PIHST07 and PIHST08 are 97.5, 91.8, 86.3, 79.6, 73.8, 69.2, 63.4 and 58.1 × 10^−6^/K, respectively. These results indicate that TECs of the PIHSTs are associated with their chemical structures.

TEC of commercially available HSTs underwent a mild increase with the increase of temperature until *T*_*m*_, and then experienced a sudden increase at temperatures higher than *T*_*m*_^51^. It is observed that TEC of PIHST increased mildly with the increase of temperature in glassy state and then underwent a sharp increase in glass transition region. It is well-known that glass transition is a thermally reversible transition corresponding to the short-range segmental motions of polymers^52^,^53^,^54^. The predominant increment in TEC at glass transition is consistent with both the amorphous nature of PIHSTs and the substantial increase in the number of degrees of freedom due to the onset of large scale of segmental motions.

### Low-temperature resistant property of PIHST

Besides the high *T*_*s*_s, application fields of high temperature HSTs are also related with the low temperatures that they can withstand^1^,^2^,^3^. For commercially available HSTs, the low temperature limits of polyolefin, polyvinylidene fluoride, silicone rubber and polytetrafluoroethylene are −45, −55, −75 and −80 °C, respectively^7^,^8^,^9^. For the PIHSTs, their shapes and heat-shrinkable properties were not affected after being stored in liquid nitrogen of −196 °C for 7 days, as shown in the supporting information (Fig. S9). The PIHSTs can withstand the low temperature of −196 °C, independent of the stress or thermal history before being immersed in liquid nitrogen.

These results indicate that the low temperature limit of PIHSTs is much lower than those of commercially available HSTs, which will extend the application fields of HSTs greatly.

### Functionality of PIHST as reducer couplings

**Figure 8:**
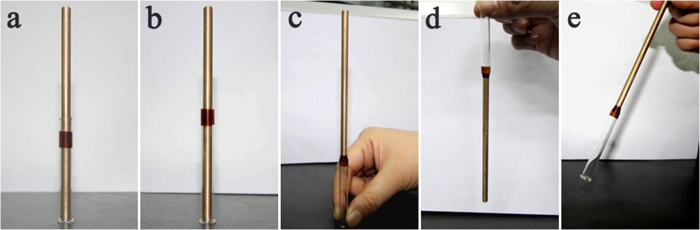
Functionality of PIHST as reducer couplings. (**a**) Expanded PIHST04 embracing a glass tube, (**b**) part of PIHST04 suspended in the air, (**c**) acting as reducer coupling with copper bar positioned vertically upward, (**d**) vertically downward and (**e**) obliquely upward.

Reducer couplings can connect and fix subjects of different sizes, and thus the combination of reducer coupling effect will further extend the application fields of HSTs. The PIHSTs also possess the functionality of reducer couplings besides the common heat-shrinkable properties, as demonstrated in Fig. 8.

The functionality of PIHT as reducer coupling was executed by embracing a glass tube tightly with the heat-shrinkage of expanded PIHST04, and a copper bar of smaller diameter was employed as the other subject to be fixed (Fig. 8a). Then PIHST04 was moved along the glass tube until its half was suspended in the air (Fig. 8b). One end of the copper bar was laid inside the expanded PIHST04 but outside the glass tube, and then they were subjected to high temperature. The heat-shrinkage of PIHST04 wrapped the copper bar tightly, thus connecting the two subjects of different sizes. At room temperature, PIHST04 become rigid and then fixed the glass tube and copper bar. The images of PIHST04 acting as reducer coupling with the copper bar positioned vertically upward, vertically downward and obliquely upward are manifested in Fig. 8c–e, respectively.

**Figure 9:**
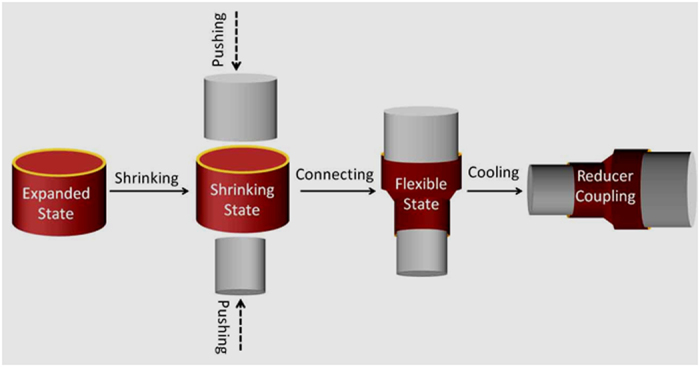
Schematic illustration of reducer coupling effect of PIHST.

The commercially available HSTs are unable to act as reducer couplings since they are flexible or semi-rigid, while the PIHSTs possess the functionality as reducer couplings since they are rigid at room temperature. When subjects smaller than the expanded but larger than the shrunk diameters of the PIHST are placed inside the expanded PIHST, the shrinkage at *T*_*s*_ will embrace the subjects and connect them. The PIHST is flexible at *T*_*s*_ but it will become rigid and then fix the subjects like a reducer coupling upon cooling with the cooling temperature of 25 °C, and the schematic illustration of its reducer coupling effect is manifested in Fig. 9.

The weight of the copper bar fixed by PIHST04 is 21.2358 g, which is more than 800 times heavier than that of PIHST04 itself (0.0265 g). Therefore, its functionality as reducer coupling is especially useful for some delicate and special devices, as the supporting frames can be spared by using the PIHSTs.

## Discussions

Rigid heat-shrinkable tubes have been prepared with shape memory polyimides, and these polyimides are soluble in organic solvents such as NMP due to the flexibility of their molecular main chains^17^,^18^,^19^,^20^. The external diameter of PIHST can be controlled by adjusting the inner diameter of the glass tubes employed as moldings, while its wall thickness can be regulated by the solution concentration and solvent evaporating speed. The PIHST possesses controllable high *T*_*g*_ from 182 to 295 °C, and *T*_*g*_ + 20 °C is regarded as its *T*_*s*_ since it can shrink back to original size quickly at this temperature. The PIHSTs with different new high *T*_*s*_s can extend the application areas of HSTs enormously, as high temperature HSTs are still lacking so far. They can withstand the low temperature of −196 °C, and the low-temperature resistant property is caused by their molecular structures, as polyimide can withstand extremely low temperatures^12^.

From the view point of microstructures, *T*_*s*_ of PIHSTs is different from that of commercially available HSTs. The temporary expanded shape of commercially available HST is fixed due to the formation of stable and less stable crystalline domains that act as reversible phase^39^. The melting of crystallites is a first-order phase transition and the restricted mobile chains in expanded tube relax and recoil during the melting process, thus endowing the HSTs with shape memory effects. Finally, a shrunken tube with permanent shape can be obtained^41^. It has been reported that the completes heat-shrinkage of commercially available HST usually occurs at temperatures slightly higher than its *T*_*m*_, and it takes less time for the HST to shrink back to its permanent shape at higher heating temperature^38^.

PIHSTs are made of amorphous shape memory polyimides, and the nice fixity of expanded diameter is mainly determined by the large difference in storage modulus at rubbery and glassy states. The PIHST was expanded at rubbery state (*T*_*g*_ + 20 °C) and the low *E*′ at this temperature favored expansion of the tube due to its low resistance. When PIHST was taken out of the oil bath, its temperature decreased rapidly and the molecular deformation due to uncoiling of polyimide chains caused by expansion was frozen, and the high *E*′ at glassy state benefited fixing of the expanded diameter due to the high rigidity^11^,^14^,^20^,^55^. It is observed that *E*′ at glassy state is more than one hundred times higher than that at rubbery state for all the PIHSTs, and *E*′ of PIHST04 at *T*_*g*_ − 30 °C (1853 MPa) is 280 times higher than that at *T*_*g*_ + 20 °C (6.6 MPa). Therefore, the expanded diameters of PIHSTs are well fixed.

The shrinking back to initial diameter of the PIHST is related with its permanent phase that mainly accounts for the shape recovery of SMPs. The PIHSTs are prepared with thermoplastic polyimides, and their long molecular chains will lead to massive chain entanglements that act as permanent phase^12^. Moreover, the polyimides contain large amount of benzene rings in their molecules, which will produce strong intermolecular π-π interactions that also act as permanent phase. As a result, the permanent phase of PIHST is composed of both chain entanglements and intermolecular π-π interactions. Accordingly, the PIHST showed high shape recovery rate and shrank back to its original diameter very well. It is observed that complete heat-shrinkage of PIHST appeared at temperature above its *T*_*g*_, and it also takes less time to shrink back at higher temperatures. The practical operations have demonstrated that *T*_*g*_ + 20 °C is effective for the expansion and shrinkage of PIHSTs.

The PIHSTs can embrace and connect subjects of different sizes with heat-shrinkage, then they will become rigid and fix the subjects like reducer couplings upon cooling. Different from the stationary diameters of commercially available reducer couplings^36^,^37^, the end diameters of PIHSTs can be altered from the values of shrunk to expanded state due to heat-shrinkage. The adjustable end diameters of PIHST will produce more convenience and flexibility in joining different subjects, and the reducer-coupling effect will spare some supporting frames.

The rigid PIHSTs may create new solutions to many technical problems and in some cases, may create a new market based on their unique properties.

## Methods

### Materials

Bis phenol A dianhydride (BPADA, 99%) and 1,3-bis(3-aminophenoxy)benzene (BAB, 98%) were bought from Sigma-Aldrich Co., 4,4′-oxydiphthalic anhydride (ODPA, 98%) and 4,4′-diaminodiphenyl ether (ODA, 99%) were bought from TCI, 4,4′-(hexafluoroisopropylidene) diphthalic anhydride (6FDA, 99%) and benzophenone-3,3′,4,4′-tetracarboxylic dianhydride (BTDA, 99.5%) were bought from J&K Scientific, and the reactants were used directly. Dimethylacetamide (DMAc) and N-methyl-2-pyrrolidone (NMP) were bought from Sinopharm Group Co. Ltd and dried with CaH_2_.

### Synthesis of polyimides

The homopolyimides of BPADA/BAB, ODPA/BAB and BTDA/BAB were synthesized by the following steps. 6 m mol diamine of BAB was added into DMAc and stirred under nitrogen in a flask until it was fully dissolved, then 6 m mol dianhydride of BPADA (ODPA, BTDA) was added into the BAB solution and stirred at 20 °C for 20 h to produce poly(amic acid) (PAA). After elimination of bubbles in vacuum chamber, the PAA was transferred onto clear glass. The imidization process was executed by step-wise curing of 70 °C/7 h, 120 °C /3 h, 160 °C/3 h, 190 °C/2 h and 230 °C/1 h for BPADA/BAB; 80 °C/7 h, 130 °C /3 h, 160 °C/2 h, 200 °C/2 h and 240 °C/1 h for ODPA/BAB; 70 °C/7 h, 120 °C /3 h, 160 °C/2 h, 210 °C/2 h and 260 °C/1 h for BTDA/BAB, respectively.

The copolyimides of 6FDA/ODA + BAB were synthesized as follows. 5 m mol ODA and BAB blends were added into DMAc and stirred under nitrogen in a flask until they were fully dissolved, then 5 m mol 6FDA was added into the diamine blends and stirred for 22 h at 20 °C to produce PAAs. After removal of bubbles, the PAA was transferred onto glass substrate and underwent imidization process of 80 °C/7 h, 120 °C /3 h, 160 °C/2 h, 200 °C/2 h, 250 °C/2 h, and 290 °C/1 h.

The polyimide films were detached away from glass substrates in water and then dried in the oven at 120 °C.

### Structural, thermomechanical and thermal characterizations

Structures of the polyimides were characterized with infrared (IR) on Thermo Nicolet Nexus 870 in the range of 600 to 4000 cm^−1^ with the spectral resolution of 1 cm^−1^.

Thermomechanical properties of the polyimides were characterized with dynamic mechanical analysis (DMA) on TA-Q800 instrument in tensile mode at the frequency of 1 Hz, and the characterization was performed on uniform specimen of 39 × 3 × 0.13 mm with heating rate of 3 °C/min.

Thermal stability was characterized with thermal gravimetric analysis (TGA) on Mettler-Toledo TGA/SDTA851 at the heating rate of 10 °C/min under nitrogen.

Differential scanning calorimetry (DSC) was characterized from room temperature to 360 °C with TA Instruments Q20 at a heating rate of 10 °C/min under nitrogen atmosphere. The sample weight was about 6 mg, and the midpoint of heat flow changes was employed as *T*_*g*_.

### Thermal expansion coefficient characterization

Thermal expansion coefficient (TEC) was characterized with thermomechanical analysis (TMA) on TA instruments Q400, and the initial length of the polyimide sample between the two grips is 15 mm. A first heating to 120 °C was executed to remove the influence of absorbed water, and changes in length of the sample were measured during the second heating with a reheating rate of 5 °C/min in nitrogen atmosphere under a static load of 0.02 N.

### Common shape memory characterization

Bending deformation was employed to examine common shape memory performance of the polyimide. The polyimide film was bended into U shape on a hot-stage set at *T*_*g*_ + 20 °C, and then the temporary shape was fixed at room temperature. When it was reheated on the hot-stage, the U shape recovered to its initial flat shape.

### Preparation of PIHSTs

After being dissolved in NMP, the polyimide solution was added into glass tubes and then placed in the high temperature oven. When the solvent was volatilized, PIHSTs clinging to the inner walls of the glass tubes were obtained.

### Heat-shrinkage characterization of PIHSTs

The PIHST was dilated in oil-bath set at *T*_*g*_ + 20 °C, and the expanded diameter was maintained at room temperature. When the expanded PIHST was placed back to the oil-bath, it would shrink back to its original size.

### Low temperature resistant characterization

Liquid nitrogen with the temperature of −196 °C was employed as low-temperature source, and PIHSTs were immersed in the liquid nitrogen for 7 days. Then they were taken out from the liquid nitrogen and characterized.

### Reducer coupling effect characterization

The expanded PIHST was attached to a glass tube with its half suspended in the air, and a copper bar was placed inside the PIHST. When they were heated, heat-shrinkage of the PIHST connected the copper bar with the glass tube. When cooled, the PIHST fixed the two subjects of different sizes like a reducer coupling.

## Additional Information

**How to cite this article**: Kong, D. and Xiao, X. Rigid High Temperature Heat-Shrinkable Polyimide Tubes with Functionality as Reducer Couplings. *Sci. Rep.* **7**, 44936; doi: 10.1038/srep44936 (2017).

**Publisher's note:** Springer Nature remains neutral with regard to jurisdictional claims in published maps and institutional affiliations.

## Supplementary Material

Supplementary Information

## Figures and Tables

**Table 1:** Physical properties of the PIHSTs.

Title	*E*′_*high*_ (MPa) at *T*_*g*_ − 30 °C	*E*′_*low*_ (MPa) at *T*_*g*_ + 20 °C	Shape fixity *(R*_*f*_, %)	Shape recovery *(R*_*r*_, %)
PIHST01	1996	6.1	98.9	99.3
PIHST02	2385	6.0	99.1	99.5
PIHST03	2228	7.2	98.7	99.2
PIHST04	1853	6.6	98.4	99.1
PIHST05	2205	8.6	98.0	98.5
PIHST06	1910	8.7	97.5	98.2
PIHST07	1769	8.9	97.0	97.8
PIHST08	1608	9.7	96.6	97.0

## References

[b1] Dinsmoor J. A. Shrinkable insulating tubing. J. Exp. Anal. Behav. 6, 170 (1963). 14027984 10.1901/jeab.1963.6-170 PMC1404272

[b2] Cook P. M. & Halperin R. M. Arthur Charlesby-his impact on creating a new industry. Radiat. Phys. Chem. 51, 7–8 (1998).

[b3] http://www.te.com/usa-en/products/heat-shrink-tubing.html.

[b4] Alzahrani E. & Welham K. Design and evaluation of synthetic silica-based monolithic materials in shrinkable tube for efficient protein extraction. Analyst 136, 4321–4327 (2011). 21863168 10.1039/c1an15447h

[b5] Tsutsumi Y., Ohashi M. & Miyoshi Y. Temperature-sensitive mechanical LPFG using contractive force of heat-shrinkable tube. Opt. Fiber. Technol. 19, 55–59 (2013).

[b6] Du B. X. & Li J. Electrical and mechanical ageing behaviors of used heat-shrinkable insulation tubes. IEEE T Dielect ElIn. 21, 1875–1881 (2014).

[b7] Morshedian J., Khonakdar H. A., Mehrabzadeh M. & Eslami H. Preparation and properties of heat-shrinkable cross-linked low-density polyethylene. Adv. Polym. Tech. 22, 112–119 (2003).

[b8] Wang J. Analysis on the industry operation of heat-shrinkable materials in 2014. China Elect. Equip. Indus. 10, 48–51 (2015).

[b9] Okano Y., Kitagawa T., Shoji N., Namioka T. & Komura M. The development of an auto-sealing system using an electrically shrinkable tube under a low-pressure condition. Mater. Perform. 36, 40–44 (1997).

[b10] Hagar J. Conductive heat-shrinkable tubing for cable shielding. Wire & Cable Tech. Int. 40, 134 (2012).

[b11] Quitmann D., Reinders F. M., Heuwers B., Katzenberg F. & Tiller J. C. Programming of Shape Memory Natural Rubber for Near-Discrete Shape Transitions. ACS Appl. Mater. Interfaces. 7, 1486–1490 (2015). 25536152 10.1021/am507184c

[b12] Xiao X. L. . Shape memory polymers with high and low temperature resistant properties. Sci. Rep. 5, 14137 (2015). 26382318 10.1038/srep14137 PMC4585657

[b13] Zhao Q., Qi H. J. & Xie T. Recent Progress in Shape Memory Polymer: New Behavior, Enabling Materials, and Mechanistic Understanding. Prog. Polym. Sci. 49–50, 79–120 (2015).

[b14] Pretsch T. Triple-shape Properties of a Thermoresponsive Poly(ester urethane). Smart Mater. Struct. 19, 015006 (2010).

[b15] Shi Y., Yoonessi M. & Weiss R. A. High temperature shape memory polymers. Macromolecules 46, 4160−4167 (2013).

[b16] Hoeher R., Raidt T., Rose M., Katzenberg F. & Tiller J. C. Recoverable strain storage capacity of shape memory polyethylene. J. Polym. Sci. B: Polym. Phys. 51, 1033–1040 (2013).

[b17] Kuder I. K., Arrieta A. F., Raither W. E. & Ermanni P. Variable Stiffness Material and Structural Concepts for Morphing Applications. Prog. Aerosp. Sci. 63, 33–55 (2013).

[b18] Koerner H. . Polymer design for high temperature shape memory: Low crosslink density polyimides. Polymer 54, 391–402 (2013).

[b19] Wang Q. H. . High performance shape memory polyimides based on π–π interactions. J. Mater. Chem. A 3, 352–359 (2015).

[b20] Chung T., Rorno-Uribe A. & Mather P. T. Two-way Reversible Shape Memory in a Semicrystalline Network. Macromolecules 41, 184–192 (2008).

[b21] Behl M., Razzaq M. Y. & Lendlein A. Multifunctional shape-memory polymers. Adv. Mater. 22, 3388–3410 (2010). 20574951 10.1002/adma.200904447

[b22] Hsu L., Weder C. & Rowan S. J. Stimuli-responsive, Mechanically-adaptive Polymer Nanocomposites. J. Mater. Chem. 21, 2812–2822 (2011). 10.1021/am9006337 PMC2840258 20305827

[b23] Xiao X. L. . Shape-memory Polymers with Adjustable High Glass Transition Temperatures. Macromolecules 48, 3582–3589 (2015).

[b24] Yoonessi M. . Graphene polyimide nanocomposites; thermal, mechanical, and high-temperature shape memory effects. Acs Nano 6, 7644–7655 (2012). 22931435 10.1021/nn302871y

[b25] Ecker M. & Pretsch T. Novel Design Approaches for Multifunctional Information Carriers. RSC Adv. 4, 46680–46688 (2014).

[b26] Jin X. D., Ni Q. Q. & Natsuki T. Composites of Multi-walled Carbon Nanotubes and Shape Memory Polyurethane for Electromagnetic Interference Shielding. J. Comp. Mat. 45, 2547–2554 (2011).

[b27] Hasan S. M., Nash L. D. & Maitland D. J. Porous Shape Memory Polymers: Design and Applications, J. Polym. Sci. Pol. Phys. 54, 1300–1318 (2016).

[b28] Hu J., Zhu Y., Huang H. & Lu J. Recent advances in shape–memory polymers: structure, mechanism, functionality, modeling and applications. Prog. Polym. Sci. 37, 1720–1763 (2012).

[b29] Yu K., Ge Q. & Qi H. Reduced time as a unified parameter determining fixity and free recovery of shape memory polymers. Nat. Comm. 5, 3066 (2014) 10.1038/ncomms4066 24423789

[b30] Kratz K., Madbouly S. A., Wagermaier W. & Lendlein A. Temperature memory polymer networks with crystallizable controlling units. Adv. Mater. 23, 4058–4062 (2011). 21815223 10.1002/adma.201102225

[b31] Xu H. X. . Deformable, Programmable, and Shape-Memorizing Micro-Optics. Adv. Funct. Mater. 23, 3299–3306 (2013).

[b32] Lendlein A. & Langer R. Biodegradable, Elastic Shape-memory Polymers for Potential Biomedical Applications. Science 96, 1673–1676 (2002). 10.1126/science.1066102 11976407

[b33] Yi D. H., Yoo H. J., Mahapatra S. S., Kim Y. A. & Cho J. W. The Synergistic Effect of the Combined Thin Multi-walled Carbon Nanotubes and Reduced Graphene Oxides on Photothermally Actuated Shape Memory Polyurethane Composites. J. Col. Inter. Sci. 432, 128–134 (2014). 10.1016/j.jcis.2014.06.060 25086386

[b34] Safranski D. L., Smith K. E. & Gall K. Mechanical Requirements of Shape-memory Polymers in Biomedical Devices. Polym. Rev. 53, 76–91 (2013).

[b35] Heuwers B., Beckel A., Krieger A., Katzenberg F. & Tiller J. C. Shape-memory natural rubber: An exceptional material for strain and energy storage. Macromol. Chem. Phys. 214, 912–923 (2013).

[b36] Datta S. K., Chaki T. K., Tikku V. K., Pradhan N. K. & Bhowmick A. K. Heat Shrinkage of Electron Beam Modified EVA. Radiat. Phys. Chem. 50, 399–405 (1997).

[b37] Khonakdar H. A., Morshedian J., Mehrabzadeh M., Wagenknecht U. & Jafari S. H. Thermal and shrinkage behaviour of stretched peroxide-crosslinked high-density polyethylene. Eur. Polym. J. 39, 1729–1734 (2003).

[b38] Yamasaki S. . Nano-Composite Heat-Shrinkable Tubing. SEI Tech. Rev. 78, 68–72 (2014).

[b39] Wang F. F. . Molecular origin of the shape memory properties of heat-shrink crosslinked polymers as revealed by solid-state NMR. Polymer 107, 61–70 (2016).

[b40] Wienhold P. D. . The development of high-temperature solar array substrate panels for the messenger spacecraft. Sampe 39, 6–17 (2003).

[b41] Dutta P., Khan M. R., Akanda M. A. S. & Uddin M. W. Stability Analysis of a Toroidal Pipe-reducer under Uniform External Pressure. Int. J. Pres. Ves. & Piping 72, 203–218 (1997).

[b42] Bloch H. P. Piping, Stationary Seals, and Gasketing, in Pump Wisdom: Problem Solving for Operators and Specialists, John Wiley & Sons, Inc., Hoboken, NJ, USA (2011).

[b43] Xiao X. L. . Optically transparent high temperature shape memory polymers. Soft Matter 12, 2894–2900 (2016). 26686222 10.1039/c5sm02703a

[b44] Chung H., Jang W., Hwang J. & Han H. Analysis of Dimensionally Stable Copolyimide with a Low-Level Residual Stress, J. Polym. Sci.B: Polym. Phy. 39, 796–810 (2001).

[b45] Han Y., Fang X. Z. & Zuo X. X. Melt processable homo- and copolyimides with high thermo-oxidative stability as derived from mixed thioetherdiphthalic anhydride isomers. Express Polym. Lett. 4, 712–722 (2010).

[b46] Zhang M. Y. . Preparation of High Performance Copolyimide Fibers via Increasing Draw Ratios, Macromol. Mater. Eng. 300, 1096–1107 (2015).

[b47] Kim S. K., Wang X., Ando S. & Wang X. G. Highly transparent triethoxysilane-terminated copolyimide and its SiO_2_ composite with enhanced thermal stability and reduced thermal expansion. Eur. Polym. J. 64, 206–214 (2015).

[b48] Kwei T. K. The Effect of Hydrogen Bonding on the Glass Transition Temperature of Polymer Mixtures. J. Polym. Sci. Lett. 22, 307–313 (1984).

[b49] Brostow W., Chiu R., Kalogeras I. M. & Vassilikou–Dova A. Prediction of Glass Transition Temperatures: Binary Blends and Copolymers. Mater. Lett. 62, 3152–3155 (2008).

[b50] Hoeher R., Raidt T., Novak N., Katzenberg F. & Tiller J. C. Shape-memory PVDF exhibiting switchable piezoelectricity. Macromol. Rapid Commun. 36, 2042−2046 (2015). 26332996 10.1002/marc.201500410

[b51] Kardos J. L., Raisoni J., Piccarolo S. & Halpin J. C. Prediction and measurement of the thermal expansion coefficient of crystalline polymers. Polym. Eng. Sci. 19, 1000–1009 (1979).

[b52] Page K. A., Cable K. M. & Moore R. B. Molecular origins of the thermal transitions and dynamic mechanical relaxations in perfluorosulfonate ionomers. Macromolecules 38, 6472–6484 (2005).

[b53] Kalogeras I. M. & Hagg Lobland H. E. The nature of the glassy state: Structure and transitions. J. Mater.Ed. 34, 69–94 (2012).

[b54] Xie T., Tunable polymer multi-shape memory effect. Nature. 464, 267–270 (2010). 20220846 10.1038/nature08863

[b55] Brostow W., Hagg Lobland H. E. & Khoja S. Brittleness and toughness of polymers and other materials. Mater. Lett. 159, 478–480 (2015).

