# Supplementary material for: Rigid High Temperature Heat-Shrinkable Polyimide Tubes with Functionality as Reducer Couplings
Source: Sci Rep. 2017 Mar 20;7:44936. doi: 10.1038/srep44936 (PMC5357908; doi:10.1038/srep44936)
Supplement: Supplementary Information [file srep44936-s1.doc]

Supplementary Information

**Rigid High Temperature Heat-Shrinkable Polyimide Tubes with Functionality as Reducer Couplings**

Deyan Kong, Xinli Xiao*

MIIT Key Laboratory of Critical Materials Technology for New Energy Conversion and Storage, School of Chemistry and Chemical Engineering, Harbin Institute of Technology, No.92 West Dazhi Street, Harbin 150001, PRC.

To whom correspondence should be addressed: X. L. Xiao (xiaoxinli@hit.edu.cn).

The polymerization process of the polyimides for PIHSTs are manifested in Figure S1 to Figure S4. The characteristic IR peaks of polyimide such as stretching vibration of C-N-C (about1372 cm^-1^), symmetric stretching of C=O (about 1717 cm^-1^) and asymmetric stretching of C=O (about 1780 cm^-1^) are observed (Figure S5a). Both the peaks of isoimide (about 1810 cm^-1^) and inter-molecular imide linkage (about 1670 cm^-1^) are absent (Figure S5b), indicating the high imidization of the PIHSTs. *T_g_s* of 6FDA/BAB+ODA copolyimides and their relationship with contents are shown in Figure S6, and TGA spectra of the PIHSTs are shown in Figure S7. The polyimides for PIHSTs showed excellent shape memory performances during common process such as bending deformation, as shown in Figure S8. The PIHSTs are low-temperature resistant and there is no change in shape and heat-shrinkable properties after they were stored in -196 °C for 7 days, as shown in Figure S9.

**Figure S1. Polymerization process of the homopolyimide for PIHST01.**

**Figure S2. Polymerization process of the homopolyimide for PIHST02.**

**Figure S3. Polymerization process of the homopolyimide for PIHST03.**

**Figure S4. Polymerization process of the copolyimides for PIHST04-PIHST08.**


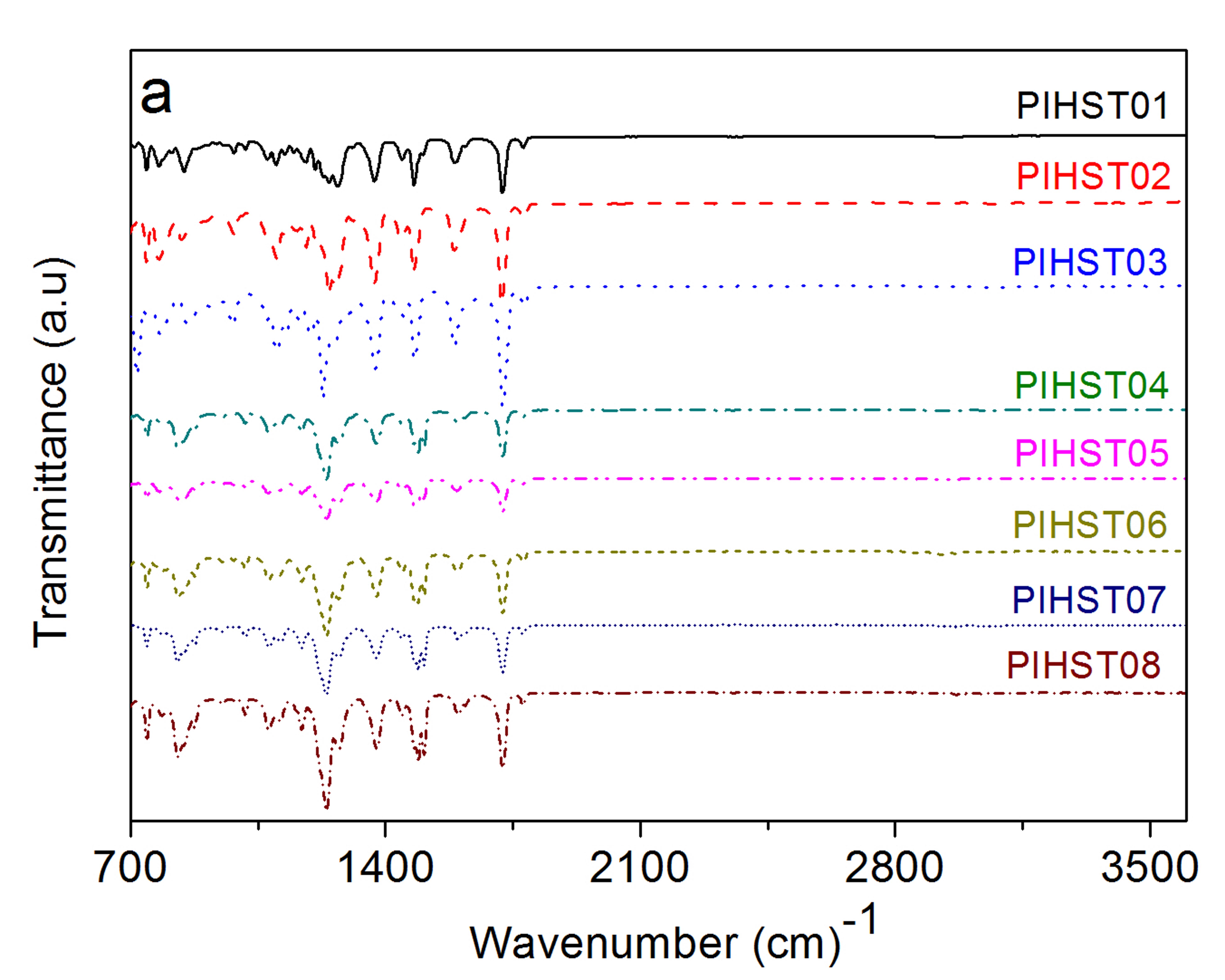

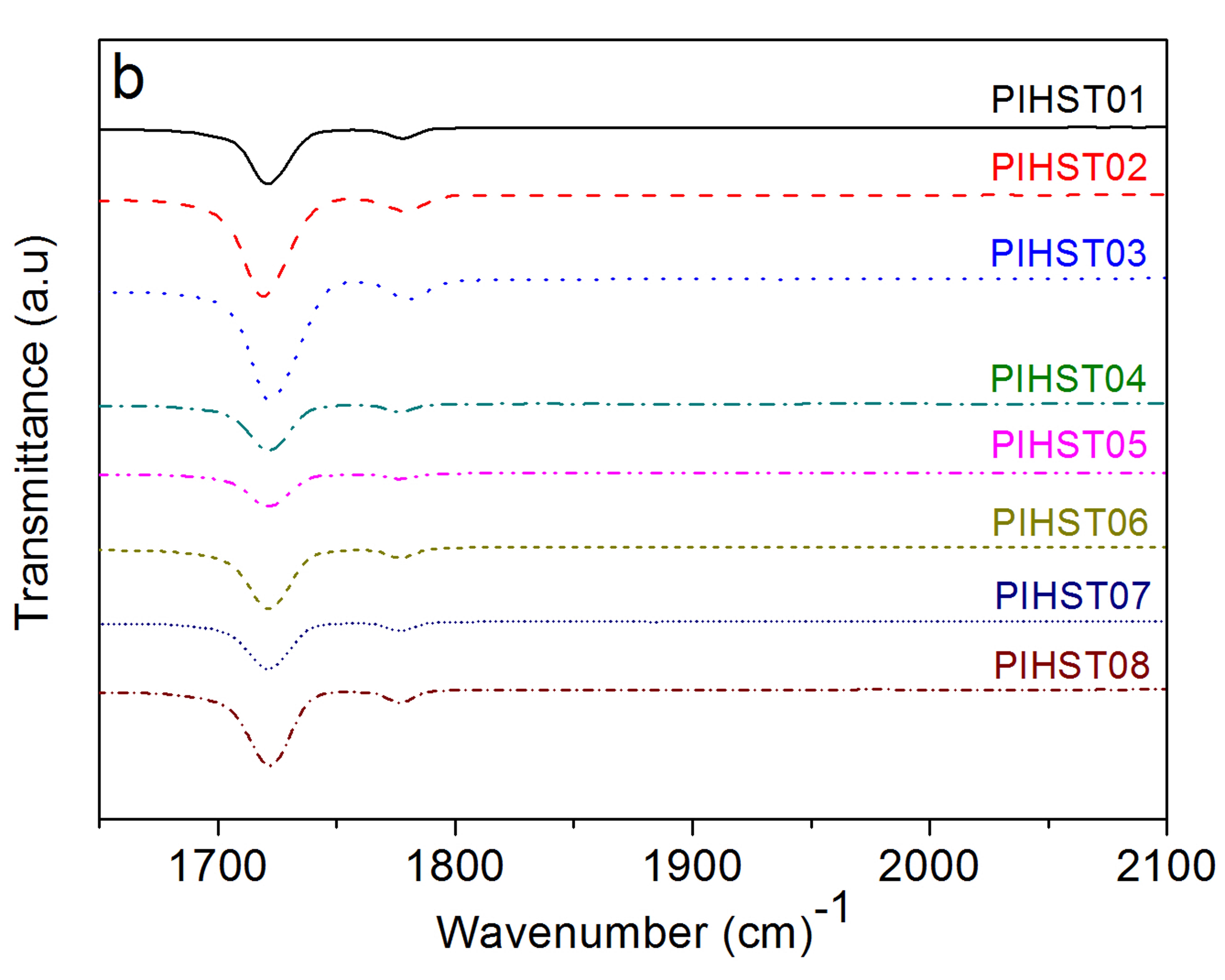


**Figure S5. IR spectra of PIHSTs.** (a) 700-3600 cm^-1^ and (b) zoom-in of 1650-2100 cm^-1^.


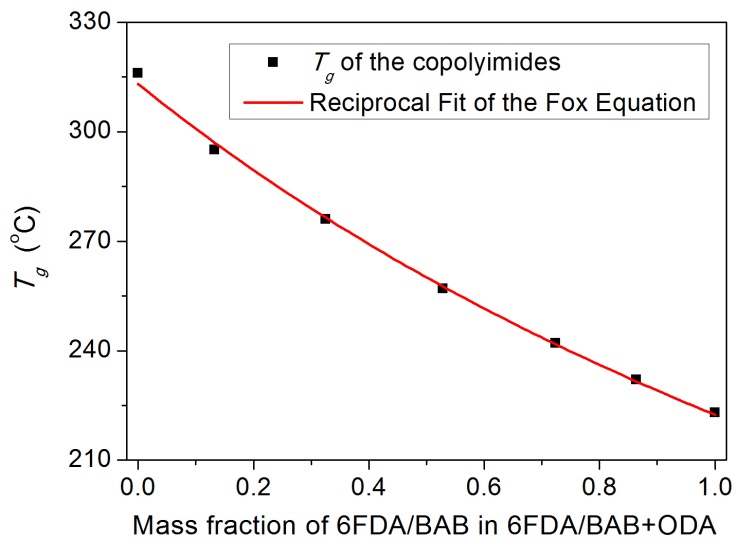


**Figure S6. *T_g_s* of 6FDA/BAB+ODA copolyimides and their relationship with contents.**


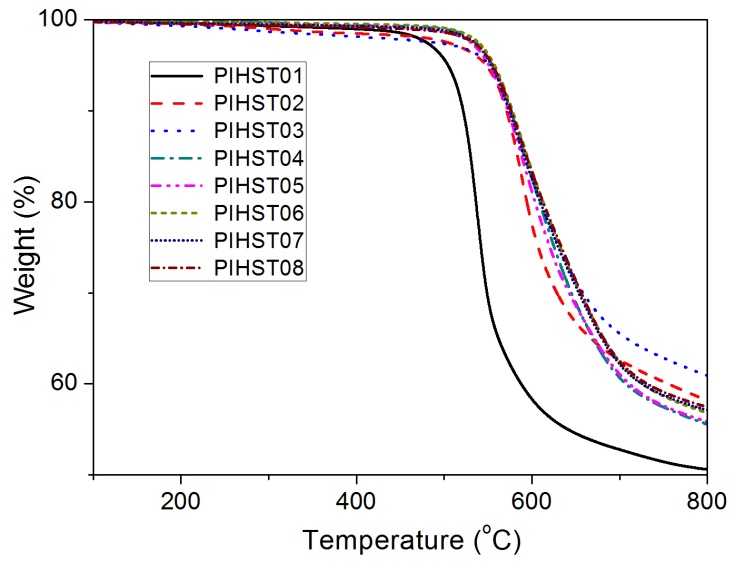


**Figure S7.** **TGA spectra of the PIHSTs.**


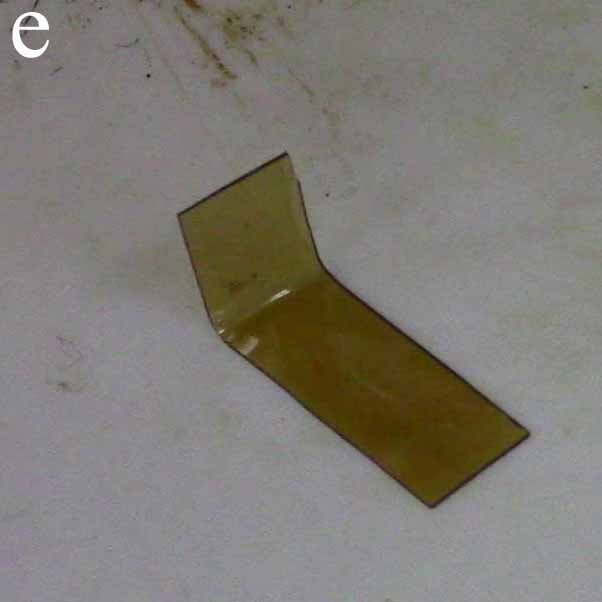

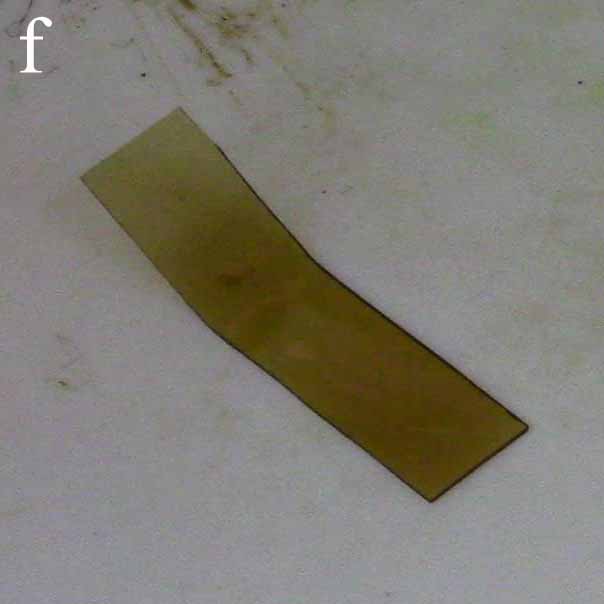

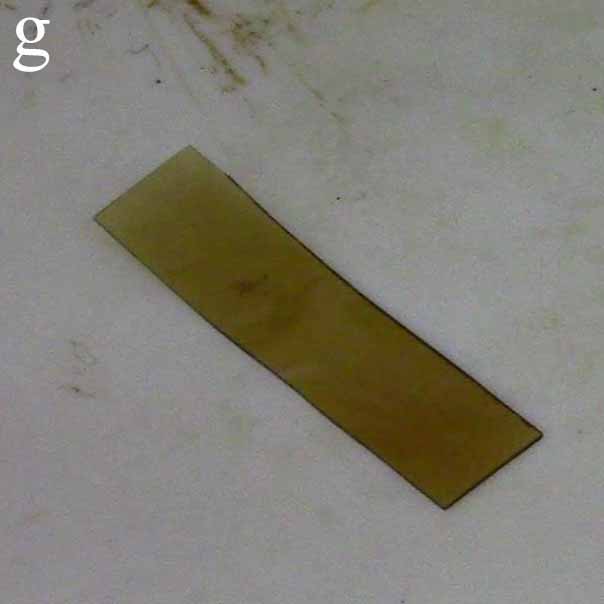

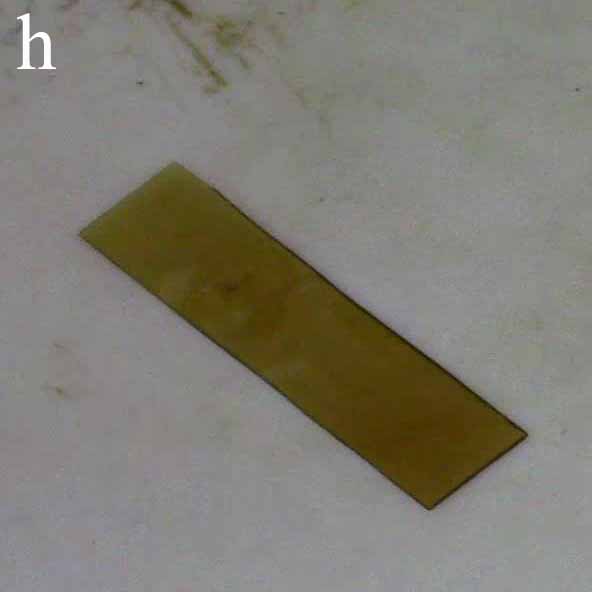

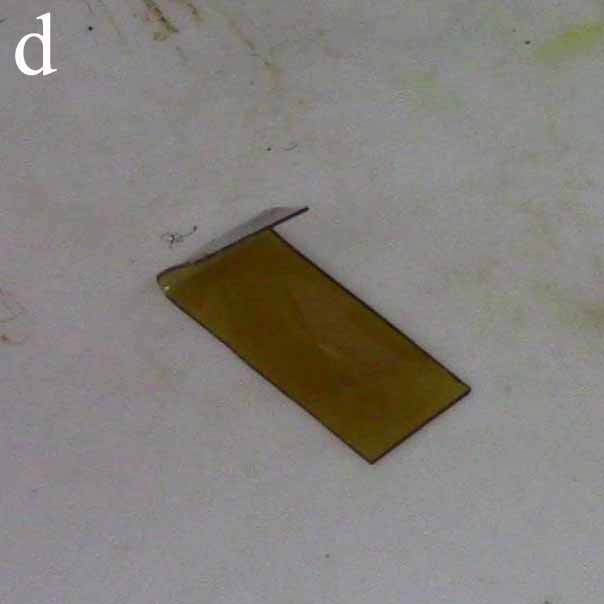

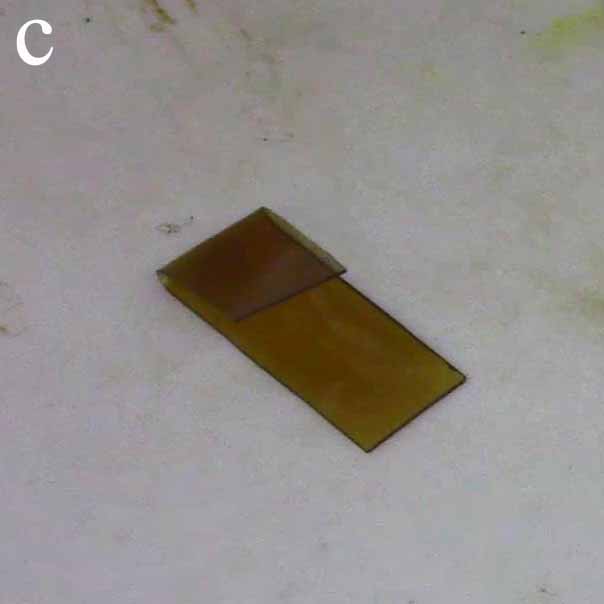

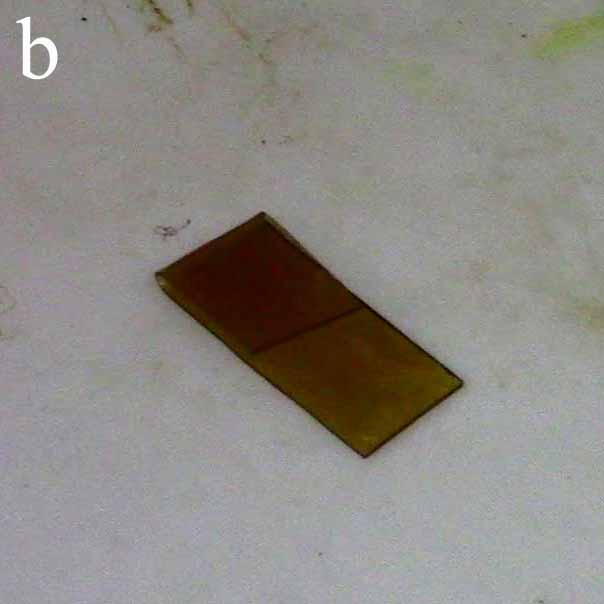

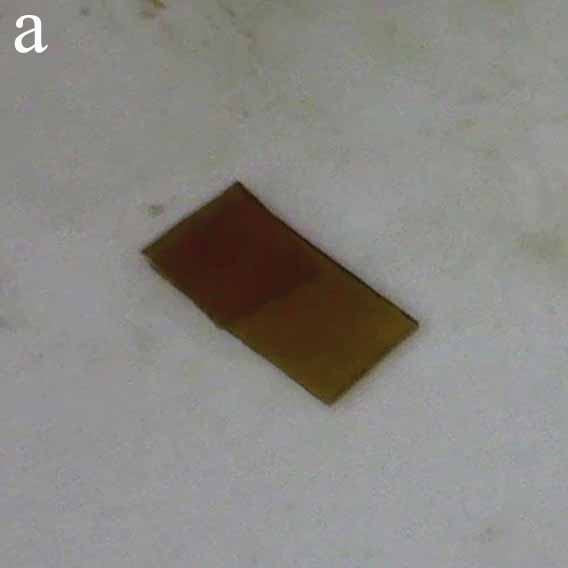


**Figure S8. Shape recovery process from bending deformation of polyimide for PIHST04 at its *T_g_*.** (a) 0 s, (b) 2 s , (c) 6 s, (d) 8 s, (e) 12 s, (f) 21 s, (g) 25 s, and (h) 28 s.


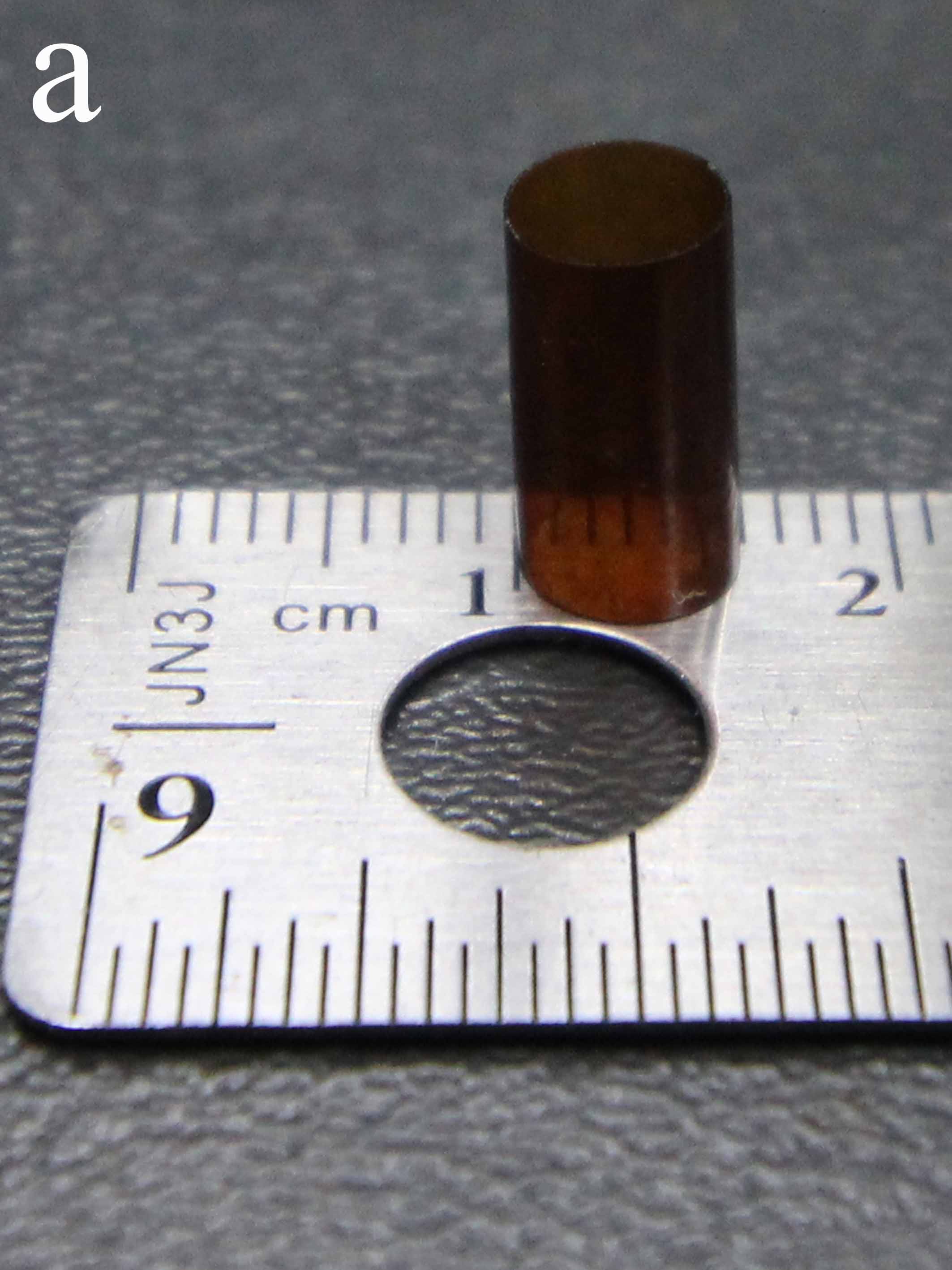

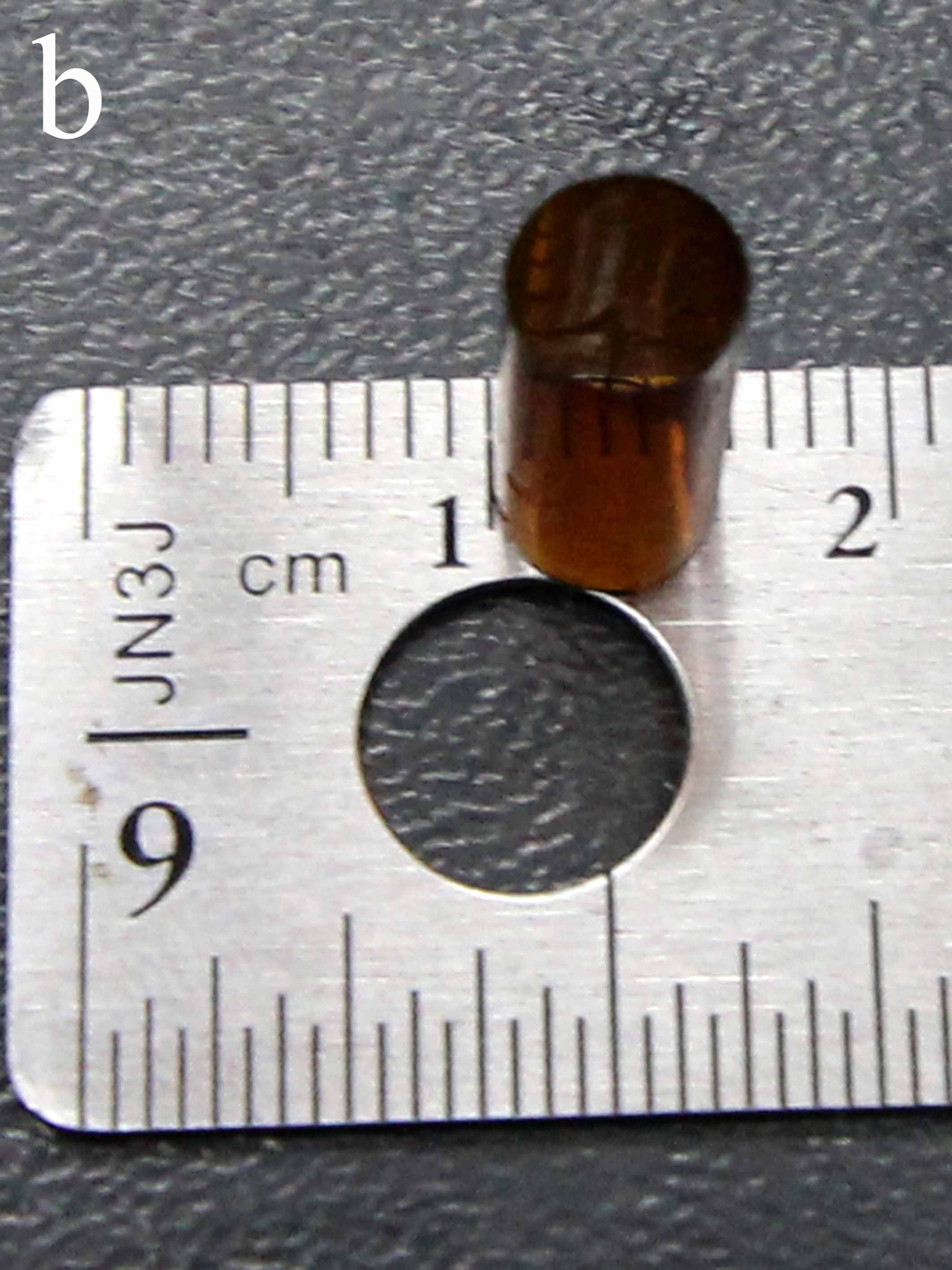


**Figure S9. Low-temperature resistant property of PIHSTs.** (a) Initial PIHST04, (b) stored in -196 °C for 7 days.
